# Supplementary material for: A systematic review of military-to-civilian transition, The role of gender
Source: PLoS One. 2025 Feb 3;20(2):e0316448. doi: 10.1371/journal.pone.0316448 (PMC11790093; doi:10.1371/journal.pone.0316448)
Supplement: Attachment — Submitted filename: Response_to_Reviewers.docx [file pone.0316448.s006.docx]

**Detailed Responses to Reviewers' Comments PONE-D-24-15468**

**Grammar, syntax, and formatting:**

1. Insert page and line numbers (continuous) into the manuscript.
2. Correct grammatical, syntax, and formatting errors.
3. Ensure consistency in terminology/references (e.g., "gender discrimination" vs "gender-based discrimination").
4. Review reference formatting and font use.

*Response:*

1. Page and line numbers have been inserted continuously throughout the manuscript.
2. All grammatical, syntax, and formatting errors have been corrected.
3. Terminology and references have been standardized throughout the document (e.g., consistent use of "gender-based discrimination").
4. Reference formatting has been reviewed and corrected, and font usage is now consistent throughout the manuscript.

**Introduction:**

1. Expand on the importance of military-civilian transition in military and veteran population studies.
2. Include an overview of similar studies and discuss findings/gaps not answered by previous studies.
3. Explain why the review is limited to the Five Eyes nations.

*Response:*

1. We have elaborated on the importance of military-civilian transition in military and veteran population studies. We've highlighted that the Five Eyes nations recognize their obligation to support veterans and families, ensuring they are not disadvantaged by their service. We've noted the UK's formalization of this commitment through the Armed Forces Covenant. We've emphasized that as women's representation in the military grows, understanding their unique transition challenges is critical for both supporting veterans and enhancing retention and recruitment efforts. (Line 120-126)
2. We've included an overview of similar studies, particularly previous scoping reviews focusing on quantitative studies related to physical and mental health outcomes. We clarified that our study differs by synthesizing qualitative literature examining the experience of military-to-civilian transition and the impact of gender, offering deeper insights into veterans' lived experiences. (Line 132-142)
3. We've explained that the review is limited to the Five Eyes nations due to their shared language and closely aligned intelligence and military policies, providing a cohesive context for examining veteran transition. (Line 116-118)

**Methodology:**

1. Include the study's registration number in the manuscript.
2. Clarify the databases used in the search.
3. Provide a deeper description of inclusion/exclusion criteria.
4. Expand on the framework analysis elements and explain why a seventh element was not included.
5. Describe how bias was avoided, particularly regarding the single screener and handling of discordancy between reviewers.

*Response:*

1. The study's registration number has been included (Open Science Framework registration: osf.io/5stuj). (Line 147)
2. We have clarified the databases used in the search. A multi-database search was conducted using Medline, Embase, PsycINFO, Pubmed, Global Health, Web of Science, and EBSCO. (Line 149-151)
3. We provided a deeper description of inclusion/exclusion criteria, detailing participant requirements, study types, and reasons for focusing on FVEY nations. (Line 160-172)
4. We explained that the transcription of verbal data into text was bypassed as we were extracting narratives from peer-reviewed studies. (Line 208-209)
5. We described our approach to avoid bias: initial screening by one reviewer using a conservative approach, followed by full-text reviews conducted independently by two reviewers with consensus required for study inclusion. (Line 176-182)

**Results:**

1. Clarify the difference between "results" and "study findings" sections or restructure headers.
2. Expand on how the "In-Service Military Experience" section contributes to the study's transition objectives.
3. Provide an expanded discussion of qualitative appraisal and thematic information, including inter-nation differences.

*Response:*

1. We altered the headings of the results to include “descriptive results” in which we discuss the characteristics of included studies, such as date of publication, country, number of participants, analytic framework etc. “Study findings” was changed to “Synthesis of findings” to indicate reporting on the themes from the included studies. (Line 222, Line 271)
2. We included additional language at the start of the "In-Service Military Experience" to provide our rationale for focusing on in-service experiences. (Line 279-291)
3. We expanded the discussion of our approach to qualitative appraisal in the methods section and included a brief discussion of the international findings. (Line 224, Line 576 – 579, Line 964)

**Discussion:**

1. The discussion seems to repeat the results section.
2. Please provide an expanded discussion on whether the findings of your study confirm, support, or counter previous studies and/or findings.
3. Are there differences in transitioning populations (gender, age, race, other)?

*Response:*

1. We addressed the repeating nature of the results section. (Full alteration of discussion section, Line 446-583)
2. We integrated previous transition studies into each of the paragraphs to confirm or counter previous findings (interspersed within discussion section Line 446-583)
3. We further discussed the inability to look at race, age and other demographics in the discussion section due to lack of demographic information on participants. (Line 582-585)
